# Effect of pharmaceutical care on the treatment of COVID-19: A protocol for systematic review and meta analysis

**DOI:** 10.1097/MD.0000000000023377

**Published:** 2020-11-25

**Authors:** Jiali Niu, Hongjun Chen, Kaixia Chen, Yin Liu, Feng Ju, Ting Xue, Dengyang Yin, Chaoqun Li, Chunxia Yin, Lingyun Jiao, Guangyu Zhao, Jixun Huang

**Affiliations:** a Department of Clinical Pharmacy; b Department of Pharmacy, Jingjiang People's Hospital, the Seventh Affiliated Hospital of Yangzhou University, Jingjiang, Jiangsu, China.

**Keywords:** coronavirus disease 2019, meta analysis, pharmaceutical care

## Abstract

**Background:**

We aimed to conduct a meta-analysis to assess the effect of pharmaceutical care on the treatment of coronavirus disease 2019 (COVID-19).

**Methods:**

All case-controlled studies related to pharmaceutical care on the treatment of COVID-19 will be included in this review. We will use index words related to pharmaceutical care and COVID-19 to perform literature searches in PubMed, Embase, MEDLINE, CNKI, and Wanfang databases, to include articles indexed as of October 20, 2020 in English and Chinese language. Two reviewers will select trials independently for inclusion and assess trial quality. Two pairs of review authors will independently extract information for each included trials. Primary outcomes are clinical outcomes, average hospital stays, costs, patient satisfaction, and incidence of adverse drug reactions. We will evaluate the risk of bias of the included studies based on Cochrane assessment tool. Revman 5.3 (the Cochrane collaboration, Oxford, UK) will be used for heterogeneity assessment, generating funnel-plots, data synthesis, subgroup analysis, and sensitivity analysis.

**Results:**

We will provide targeted and practical results assessing the effect of pharmaceutical care on the treatment of COVID-19.

**Conclusion:**

The stronger evidence about the effect of pharmaceutical care on the treatment of COVID-19 will be provided for clinicians.

**Systematic review registration number:**

PROSPERO CRD42020214223

**Ethics and dissemination:**

There is no need for ethical approval, and the review will be reported in a peer-reviewed journal.

## 1 Introduction

The novel coronavirus disease 2019 (COVID-19) is caused by infection from the newly emerged, highly contagious severe acute respiratory syndrome coronavirus 2 (SARS-CoV-2).^[1]^ SARS-CoV-2 mainly invades the respiratory tract and lungs and severe cases of COVID-19 can progress rapidly to septic shock, acute respiratory distress syndrome, and multiple organ dysfunction syndrome.^[2]^ Many academic institutions and research groups are working collaboratively on beneficial interventions.^[3]^ There are numerous drugs for the treatment of COVID-19, but there is few widely effective drug therapy and many drugs are still only experimental. Timely evaluation and monitoring of drug treatment is particularly important for patients with COVID-19.

Pharmaceutical care is provided by clinical pharmacists to promote health, wellness, and disease prevention.^[4]^ At present, an increasing number of studies have evaluated the value of clinical pharmacist participation in clinical work and have confirmed that clinical pharmacists can regulate the use of antibiotics,^[5]^ reduce adverse drug reactions (ADRs), and even reduce treatment costs.^[6]^ To date, the effect of pharmaceutical care on the treatment of COVID-19 has been investigated in several studies^[7,8]^ and found that pharmaceutical care was benefit for clinical treatment. To provide stronger evidence for the clinical practice, we aim to conduct a meta- analysis of cohort studies to assess the effect of pharmaceutical care on the treatment of COVID-19.

## 2 Methods

### 2.1 Registration

This protocol has been registered with the International Prospective Register of Systematic Reviews in October 20 as CRD42020214223. In this paper, the protocol will be performed according to the Preferred Reporting Items for Systematic Review and Meta-Analysis Protocols guidance^[9,10]^ and Cochrane Handbook for Systematic Reviews of Intervention. If we will refine procedures described in this protocol, we will document the amendments in the International Prospective Register of Systematic Reviews database and disclose them in future publications related to this meta-analysis.

### 2.2 Inclusion criteria for considering studies

#### 2.2.1 Types of studies

All case-controlled studies, comparing the prognosis of patients COVID-19 received/not received pharmaceutical care will be included in this review.

#### 2.2.2 Types of participants

The diagnosis of COVID-19 was confirmed as positive result for respiratory pathogen nucleic acid test and nasopharyngeal swab with high-throughput sequencing or real-time reverse transcriptase polymerase chain reaction.

#### 2.2.3 Types of interventions

Patients received/not received pharmaceutical care.

#### 2.2.4 Types of outcome assessments

Any available information about the effect of pharmaceutical care on the treatment of COVID-19 will be assessed. Primary outcomes are clinical outcomes, average hospital stays, costs, patient satisfaction and incidence of ADRs.

### 2.3 Search strategy

We will use index words related to pharmaceutical care and COVID-19 to perform literature searches in PubMed, Embase, MEDLINE, CNKI, and Wanfang databases, to include articles indexed as of October 20, 2020 in English and Chinese language. The key search terms will be used are [“Pharmaceutical care” or “Pharmacist” or “Clinical Pharmacists” or “Clinical Pharmacist” or “Pharmacist, Clinical” or “Pharmacists, Clinical” or “Community Pharmacists” or “Community Pharmacist” or “Pharmacist, Community” or “Pharmacists, Community” or “Retail Pharmacists” or “Pharmacist, Retail” or “Pharmacists, Retail” or “Retail Pharmacist” and “2019 novel coronavirus disease” or “COVID19” or “COVID-19 pandemic” or “SARS-CoV-2 infection” or “COVID-19 virus disease” or “2019 novel coronavirus infection” or “2019-nCoV infection” or “coronavirus disease 2019” or “coronavirus disease-19” or “2019-nCoV disease” or “COVID-19 virus infection”]

### 2.4 Data collection

#### 2.4.1 Selection of studies

Two reviewers will independently select trials for inclusion. We will exclude articals if they meet any of the following criteria:

(1) fewer than 10 patients;
(2) studies not comparing pharmaceutical care and none pharmaceutical care on the treatment of COVID-19.

**Figure 1:**
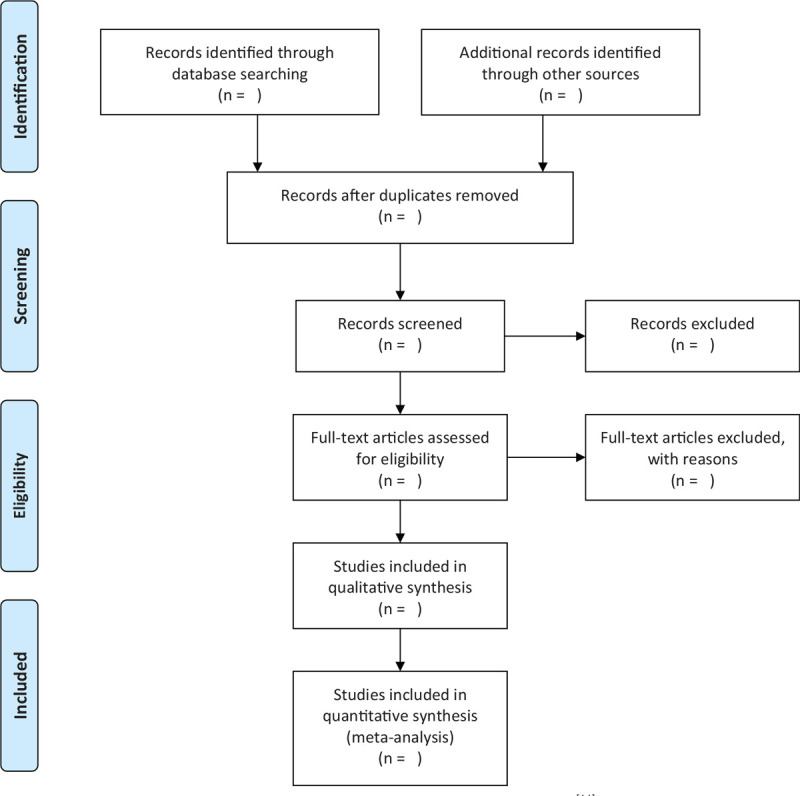
Flow diagram of the study selection process.^[11]^

The specific process of study selection is shown in Figure 1.

#### 2.4.2 Data and information extraction

Two pairs of review authors will independently extract general information for each included trial, including the name of first author, year, country, design, sample size, average age, and sex ratio. The fifth author will check all the data.

In the same manner, we will extract data for effect assessments. For each study, we will extract the following information: clinical outcomes, average hospital stay, total hospitalization cost, incidence of ADRs, and patient satisfaction. We will resolve disagreements in the numbers extracted by discussion.

### 2.5 Assessment of risk of bias

The review authors will independently assess the quality of the trials included in the review, in accordance with Chapter 8 of the Cochrane Handbook for Systematic Reviews of Interventions (Higgins 2011), by

(1) random sequence generation,
(2) allocation concealment,
(3) blinding of participants and personnel,
(4) blinding of outcome assessment,
(5) incomplete outcome data,
(6) selective reporting, and
(7) other bias.

The fifth author will check all the data. We will use this information to evaluate quality and resolve disagreements by discussion until consensus is reached.

### 2.6 Data analysis

#### 2.6.1 Assessment of heterogeneity

The Chi-squared test and I^2^ statistic will be used to assess heterogeneity. It indicates that the heterogeneity exceeds the acceptable range when *P* < .10 or I^2^ > 50%. If the heterogeneity is in the acceptable range (*P* > .10, I^2^ < 50%), the fixed effect model shall be used for data analysis; otherwise, the random effect model will be adopted.

#### 2.6.2 Date synthesis

Two pairs of review authors will independently extract information for each included trial and whether all the participants are accounted for in the analysis. The fifth author will check all the data. We will use Review Manager 5.3 to assess the risk of bias, heterogeneity, sensitivity and subgroup analysis. We will calculate a weighted estimate of the treatment effect across trials and for the interpretation of the results and we will use 95% CI. *P* < .05 will be considered statistically significant.

#### 2.6.3 Subgroup analysis

We will do the following subgroup analysis to explore the possible causes of high heterogeneity:

(1) articles with different impact factors (≥5, 3 ∼ 5, and ≤3) and
(2) trials with low and high risk of bias.

#### 2.6.4 Sensitivity analysis

We will conduct sensitivity analysis by excluding trails one by one and observe whether the synthesis result changes significantly. If there are significant changes, we will make a decision cautiously to decide whether to merge it. If the changes not significantly, it indicates that our synthesized result is firm.

### 2.7 Assessment of publication bias

If more than 10 articles are available for analysis, funnel plots will be generated to assess publication bias. A symmetrical distribution of funnel plot data indicates that there is no publication bias, otherwise, we will analyze the potential reasons for this outcome and give reasonable interpretation for asymmetric funnel plots.

### 2.8 Confidence in cumulative evidence

We will use the Grades of Recommendations Assessment, Development and Evaluation system to assess the quality of our evidence.^[10]^ According to the grading system, the level of evidence will be rated high, moderate, low and very low.

## 3 Discussion

Pharmaceutical care is the main tasks of clinical pharmacist,^[12,13]^ including

(1) participating in the formulation of clinical treatment plans;
(2) instructing physicians to use drugs rationally and putting forward suggestions for the irrational use of drugs during the treatment by consulting relevant literature;
(3) providing pharmaceutical consulting services, giving the doctor reasonable advice on the medication and treatment;
(4) evaluating the drug efficacy and ADRs;^[14,15]^ and
(5) educating patients on medication, so that patients have a clear understanding of drug effects and ADRs.^[16]^

Studies show that pharmaceutical care can promote the safety, effectiveness, rationality, and economy of drug application in clinical practice.^[17–19]^

This study will conduct a meta-analysis of related cohort studies, and provide the current evidence on the effect of pharmaceutical care on the treatment of COVID-19, so as to better guide clinical practice.

## Author contributions

**Conceptualization:** Jixun Huang, Guangyu Zhao, Hongjun Chen, Jiali Niu, Kaixia Chen, and Yin Liu.

**Funding acquisition:** Jixun Huang and Guangyu Zhao.

**Investigation:** Jiali Niu, Hongjun Chen, Guangyu Zhao, and Jixun Huang.

**Methodology:** Jiali Niu, Yin Liu, Chaoqun Li, Chunxia Yin, Lingyun Jiao, Feng Ju, Ting Xue, and Dengyang Yin.

**Software:** Jiali Niu, Hongjun Chen, Kaixia Chen, and Yin Liu.

**Supervision:** Hongjun Chen, Kaixia Chen, Yin Liu, and Guangyu Zhao.

**Writing – original draft:** Jiali Niu, Hongjun Chen, Kaixia Chen, Yin Liu, Feng Ju, Ting Xue, Dengyang Yin, Chaoqun Li, Chunxia Yin, and Lingyun Jiao.

**Writing – review & editing:** Jixun Huang and Guangyu Zhao.
